# A Multi-Scale Liver Tumor Segmentation Method Based on Residual and Hybrid Attention Enhanced Network with Contextual Integration

**DOI:** 10.3390/s24175845

**Published:** 2024-09-09

**Authors:** Liyan Sun, Linqing Jiang, Mingcong Wang, Zhenyan Wang, Yi Xin

**Affiliations:** College of Computer Science and Technology, Changchun University, No. 6543, Satellite Road, Changchun 130022, China; sunly@ccu.edu.cn (L.S.);

**Keywords:** liver and tumor segmentation, u-net, hybrid attention mechanism, multi-feature extraction

## Abstract

Liver cancer is one of the malignancies with high mortality rates worldwide, and its timely detection and accurate diagnosis are crucial for improving patient prognosis. To address the limitations of traditional image segmentation techniques and the U-Net network in capturing fine image features, this study proposes an improved model based on the U-Net architecture, named RHEU-Net. By replacing traditional convolution modules in the encoder and decoder with improved residual modules, the network’s feature extraction capabilities and gradient stability are enhanced. A Hybrid Gated Attention (HGA) module is integrated before the skip connections, enabling the parallel processing of channel and spatial attentions, optimizing the feature fusion strategy, and effectively replenishing image details. A Multi-Scale Feature Enhancement (MSFE) layer is introduced at the bottleneck, utilizing multi-scale feature extraction technology to further enhance the expression of receptive fields and contextual information, improving the overall feature representation effect. Testing on the LiTS2017 dataset demonstrated that RHEU-Net achieved Dice scores of 95.72% for liver segmentation and 70.19% for tumor segmentation. These results validate the effectiveness of RHEU-Net and underscore its potential for clinical application.

## 1. Introduction

Liver cancer, a malignant tumor with a high incidence rate, is a leading cause of cancer-related deaths worldwide, as well as in China [1,2]. Data projections indicate that the mortality rate for liver cancer will continue to increase over the next few years [3], posing a significant threat to human health and life safety. Consequently, early detection and diagnosis are imperative to significantly reducing mortality rates among patients [4]. In this context, Computed Tomography (CT) has become an essential diagnostic tool in modern medicine. CT scans use advanced X-ray technology to capture images from various angles by rotating around the patient. These images are processed by algorithms to generate detailed cross-sectional views of internal anatomy. The success of this technology depends on high-precision sensors that capture high-resolution images, enabling more accurate diagnoses and effective treatment plans. However, manually delineating liver tumor regions in these images is labor-intensive and time-consuming, often influenced by physicians’ subjective interpretations. The low contrast and complex morphology of liver tumors compared to surrounding tissues further complicate this task [5]. Rapid advancements in computer-aided diagnosis technologies are improving automatic disease identification, reducing diagnostic errors, and promoting automation in medical diagnosis. These technologies enhance efficiency and further scientific research and clinical practice [6]. Despite this progress, visual recognition of liver tumors remains challenging due to significant individual variations in liver anatomy and similar tissue densities between the liver and adjacent organs [7]. Nevertheless, automatic segmentation methods based on neural networks have proven to be efficient and accurate for segmenting liver tumors [8], highlighting the importance of ongoing research in this field for enhancing liver tumor treatments.

In the realm of medical image analysis, traditional segmentation techniques utilize image features such as color, texture, and shape. Common methods include the threshold method [9], region growing [10,11], and clustering [12,13,14]. The threshold method segments the image into different regions based on one or more predefined threshold values. Anju et al. applied an adaptive threshold method to segment abdominal CT images for extracting liver and tumor regions, which involved denoising the images and automatically adjusting thresholds based on regional characteristics [15]. The region-growing method expands the segmentation area by leveraging the similarity between seed points and neighboring pixels. Qiu et al. refined this technique by incorporating dual growth criteria and dynamic thresholds to enhance the precision of liver edge segmentation and reduce over-segmentation effectively [15]. Furthermore, Das et al. employed an adaptive threshold, morphological operations, and a nuclear fuzzy C-means clustering algorithm, integrated with spatial information, to segment liver abdominal CT images [16]. Xu et al. developed a liver tumor segmentation approach based on a multi-scale region active contour model that adaptively adjusted parameters for precise segmentation, demonstrating high precision and efficiency across various CT datasets [17]. Traditional methods are effective for organ images that display clear boundaries and high contrast. However, they often struggle with tumor images characterized by blurred boundaries or low contrast. This struggle stems from their reliance on predefined rules and a lack of adaptability. Consequently, accurate and automatic segmentation of liver and tumor regions remains challenging [18]. These limitations have prompted researchers to explore more advanced techniques that can accommodate the complexities and variability of tumor morphology.

In recent years, the application of deep learning technologies in medical image segmentation has seen substantial growth due to its exceptional capabilities [19]. The U-Net model, introduced by Ronneberger et al. [20], significantly enhances the precision of medical image segmentation through innovative skip connections and a symmetrical structure, establishing a robust foundation for further research [21]. Oktay et al. incorporated the attention mechanism into U-Net, developing the Attention U-Net, which improved the model’s ability to focus on critical regions and boosted segmentation accuracy [22]. Moreover, the Resunet++ model proposed by Jha et al., which integrates residual linkages [23], the SE module [24], and the ASPP [25], extends the context capture capability of the model, enhancing performance while also increasing its complexity.

While these models excel in complex scenarios or data-scarce environments, they still face challenges regarding generalization and performance, particularly when segmenting liver tumor images with blurred boundaries, low contrast, or small target sizes. These models often fall short in capturing detail, adaptability, and richness in feature representation. Therefore, this study aims to enhance the performance and segmentation accuracy of liver tumor segmentation algorithms based on deep learning. Medical images, particularly those of tumors, often contain structures of various sizes and shapes. As single-scale feature extraction may not effectively capture all essential details, this paper proposes an improved U-Net model. This model employs multi-scale feature extraction techniques, which enable the simultaneous gathering of information from different spatial scales. This approach more comprehensively represents image content and enhances the model’s ability to recognize and segment complex structures. Furthermore, to optimize the effect of feature fusion, the model introduces a dynamic feature fusion mechanism. Unlike traditional static feature fusion, this dynamic approach can adjust the combination weights of features at different scales based on specific tasks. This method not only considers the relative importance of features but also refines the weighting and optimization of features through learning the interactions among them. Such a design enhances both the segmentation precision and the adaptability of the model. The model integrates multi-scale feature extraction and dynamic feature fusion technologies. It enables the network to effectively learn multi-dimensional and irregular features of complex structures, demonstrating enhanced robustness and generalization performance.

## 2. Related Work

### 2.1. Multi-Scale Feature Fusion

In the field of deep learning, multi-scale feature extraction and fusion technology are the keys to improving the performance of the model [26]. By using convolution kernels of different sizes to capture information at the same time, GoogleNet enhances the expression ability and detail capture ability of the network. Spatial Pyramid Pooling (SPP) proposed by SPPNet [27] effectively avoids the distortion problem caused by image cropping and scaling and solves the problem of repeated image feature extraction by convolutional neural networks. Inspired by SPP, DeepLabV3 [28] proposes the atrous Spatial Pyramid Pooling (ASPP) module, which uses multiple parallel atrous convolutional layers with different sampling rates, and the features extracted for each sampling rate are further processed in separate branches and fused to generate the final result. This module constructs convolution kernels of different receptive fields through different void rates to obtain multi-scale object information. By constructing a multi-level pyramid, features of different resolutions are fused at each level. The pyramid’s top-down structure ensures that each level obtains rich semantic information while maintaining high-resolution details. This architecture significantly improves the detection performance of small objects.

In the process of liver tumor segmentation, the distribution of tumors is scattered, and individual differences are large, with different sizes and shapes. Inspired by the above models, the MSFE module is designed and integrated into U-Net in this study. The MSFE module expands the receptive field to enrich semantic information and effectively captures global context information by using atrous convolution with multi-level sampling rates for parallel sampling. The module alleviates the problem of local information loss that may be caused by dilated convolution by reweighting and optimizing the feature processing process. At the same time, the module suppresses irrelevant or interfering information. This suppression enriches the semantic data available during the decoding stage, thereby improving both the accuracy and generalization capability of the segmentation task. It compensates for the limitations of traditional convolution in processing tumors of varying sizes.

### 2.2. Attention Mechanism

By dynamically adjusting the feature weights in the model, the attention mechanism makes the model focus on the key information in the current task and significantly enhances the feature extraction ability. Oktay et al. introduced the attention gate into the skip connection of U-Net. By weighting the features in the skip connection, the attention gate helps the model ignore irrelevant background signals when merging low-level and high-level features and enhances the model’s control of the importance of different features [22]. The Squeeze-and-Excitation (SENet) proposed by Hu et al. enables the network to adaptively adjust the characteristic response of each channel so as to strengthen the useful features and suppress the unimportant features [24]. Based on SENet, Roy works in parallel through the spatial and channel squeeze excitation Block (scSE-block), which not only adjusts the dependence between channels but also considers the spatial correlation. It has shown good results in image segmentation tasks [29]. Woo et al. introduced the Convolutional Block Attention Module (CBAM), which combined spatial and channel attention mechanisms to adaptively adjust the weight of the feature map so that the network focused on important feature information [30]. Wang proposed a channel attention module inserted into the skip connection between the encoder and decoder, which can improve the performance of medical image segmentation [31]. The Dual Cross-Attention module addresses the semantic gap between encoder and decoder features by sequentially capturing the channel and spatial dependencies of multi-scale encoder features [32].

Inspired by the network models discussed above, this paper proposes a novel Attention module named Hybrid Gated Attention (HGA). It is integrated into U-Net, enabling the network to identify and process key features more accurately and enhance its ability to integrate contextual information. It effectively makes up for the shortcomings of traditional U-Net in low efficiency of feature selection, insufficient detail recovery, and more accurate segmentation of small tumors.

## 3. Methods

**Figure 1:**
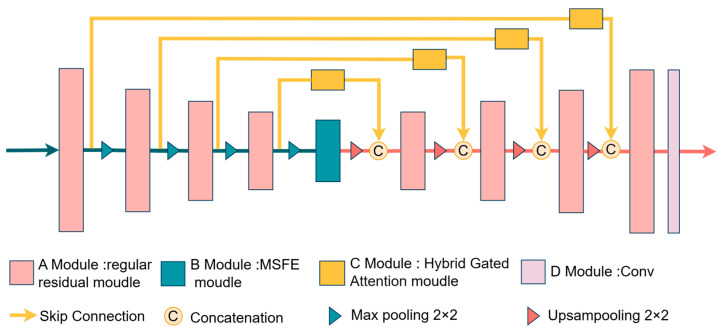
Architecture of RHEU-Net, where Module A denotes the residual module, Module B refers to the Multi-Scale Feature Enhancement module (MSFE), Module C indicates the Hybrid Gated Attention module (HGA), and Module D includes convolution operations.

This paper proposes a network called RHEU-Net, which leverages multi-scale technology and a hybrid attention mechanism to enhance its ability to extract contextual information, addressing the issue of missing small targets and unclear target boundaries in CT images. The network architecture is shown in Figure 1.

The proposed RHEU-Net essentially retains a structure similar to the traditional U-Net, but it replaces the regular convolutional blocks in the encoder and decoder with improved residual modules to preserve more low-level features while mitigating the problem of gradient vanishing. Additionally, a Hybrid Gated Attention (HGA) module is added at each skip connection. In our proposed RHEU-Net, before the feature maps from the encoder are concatenated with those from the decoder, they are first optimized by the HGA, which filters out irrelevant features. The Multi-Scale Feature Enhancement (MSFE) technique replaces the regular convolutional blocks in the bottleneck layer of the traditional U-Net. MSFE expands the model’s receptive field by using atrous convolutions with different sampling rates in parallel, thereby enriching semantic information and effectively capturing global contextual information. Additionally, the module reweights and optimizes the feature processing workflow. It provides rich semantic information for the decoding phase, thereby improving the accuracy and generalization capability of the segmentation task. This enhancement compensates for the limitations of ordinary convolution in processing tumors of varying sizes.

### 3.1. Residual Module

In deep learning, increasing the width and depth of a neural network can enhance its performance. However, adding too many layers often leads to vanishing or exploding gradients. To address this, He Kaiming et al. developed the Residual Network (ResNet), which introduces skip connections every two layers, helping the network maintain its efficacy even as the number of layers increases [33].

**Figure 2:**
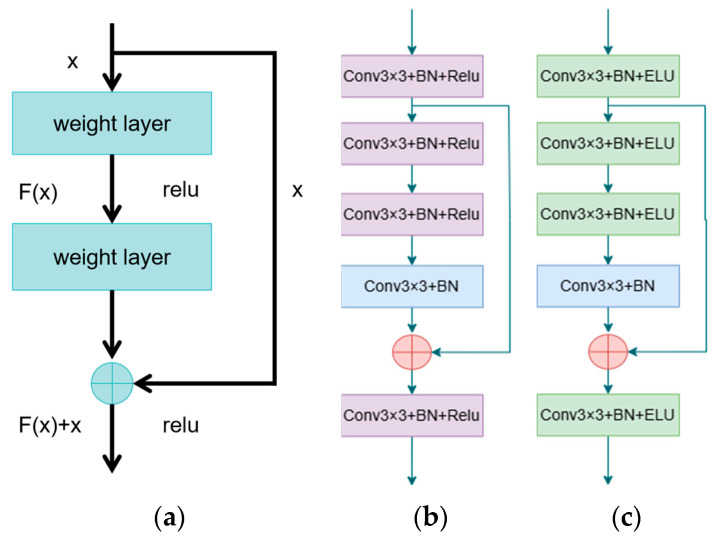
(**a**) Structure of the residual module in ResNet; (**b**) Structure of the residual module used in the encoder; (**c**) Structure of the residual module used in the decoder.

While deeper networks generally surpass shallow ones in various tasks, excessive depth can lead to performance saturation or even degradation—a phenomenon known as network degradation. ResNet combats this through its innovative use of residual blocks that employ residual learning and identity mapping, promoting higher accuracy and faster convergence [33]. As shown in Figure 2a, the formula for the residual block is expressed as follows.

(1) H(x)=F(x)+x

where H(x) is the output of the layer, x is the input feature, and F(x) represents the residual component the network learns [34]. Ideally, if the input closely matches the output, F(x) approaches zero, making H(x) nearly equivalent to x. If significant differences exist, F(x) captures these variations, adjusting the output incrementally based on x. Residual connections allow the network to modify the input rather than outputting a complete new set, easing the gradient flow back to earlier layers and reducing gradient vanishing issues while boosting training effectiveness.

Inspired by the ResNet architecture, we improve the U-Net network to enhance the learning ability and feature transfer efficiency of the network by replacing the standard double 3 × 3 convolutional blocks with improved residual modules in the upsampling and downsampling stages. In the encoder part, the modified residual module (shown in Figure 2b first processes the input through a primary convolutional block (labeled conv1, containing 3 × 3 convolutions, batch normalization (BN), and ReLU activation function). Subsequently, the deep features are further extracted by a three-segment convolutional sequence consisting of two conv1 and an intermediate conv2 (3 × 3 convolution + BN). Eventually, the output of conv1 is added to the output of conv2 to form the residual connection. The residual concatenated feature map is processed through another conv1 block to output the final feature map.

In the decoder part, the structure of the residual module (shown in Figure 2c is similar to the encoder, but the difference lies in the choice of activation function; here the ELU activation function is adopted to optimize the gradient response for negative values. The encoder primarily extracts features from the input image efficiently and reduces its dimensions, using the ReLU activation function for its computational efficiency and effectiveness in mitigating the vanishing gradient problem. Conversely, the decoder’s task is to reconstruct the input image from compressed features, aiming to preserve and recover as much of the original image’s detail as possible. The ELU activation function, which does not saturate with negative inputs and features an exponential characteristic, aids in smoothly learning and reconstructing details. This change enhances the network’s feature extraction capability and improves gradient flow, effectively addressing the vanishing gradient problem and allowing the network to retain and learn low-level features more effectively. Moreover, the flexibility in choosing activation functions further optimizes the detailed reconstruction of features, thus enhancing the model’s overall performance.

### 3.2. Hybrid Gated Attention

In the realm of medical image segmentation, attention mechanisms have been extensively employed to heighten the sensitivity of models to critical data. The traditional Squeeze-and-Excitation (SE) block employs channel-wise attention, utilizing global average pooling and fully connected layers to amplify the model’s reliance on significant channels [24]. Nevertheless, this method overlooks the spatial characteristics of images, potentially neglecting essential spatial information. To address this deficiency, the spatial and channel Squeeze-and-Excitation (scSE) block enhances the SE block’s functionality by concurrently processing spatial and channel attentions, thereby ensuring a more thorough capture of both spatial and channel information [29]. Despite these improvements, the feature fusion strategy of the scSE remains relatively static, with limited capacity for dynamic adjustment. The Convolutional Block Attention Module (CBAM) advances this concept by methodically processing channel and spatial attentions in sequence, thereby affording more precise control [30]. However, such sequential processing may lead to the permanent loss of vital information at preliminary stages.

**Figure 3:**
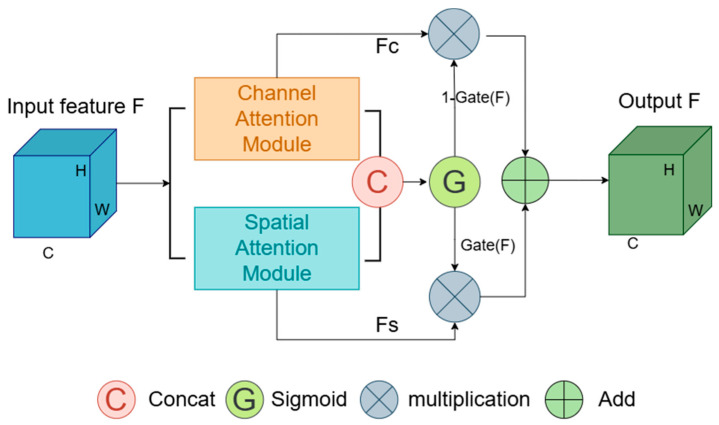
Structure of the Hybrid Gated Attention module.

Building upon these models, this study introduces a novel attention mechanism, the Hybrid Gated Attention (HGA) module, and incorporates it into the U-Net architecture. As shown in Figure 3, the HGA module processes channel and spatial attentions simultaneously and integrates a gated mechanism. This mechanism dynamically adjusts the feature fusion strategy based on the network’s real-time status and the input features’ characteristics. This capacity for dynamic adjustment enables the model to effectively learn the multi-dimensional and irregular features of complex structures. It captures crucial channel and spatial feature information during segmentation, compensates for missing details, and improves segmentation accuracy.

**Figure 4:**
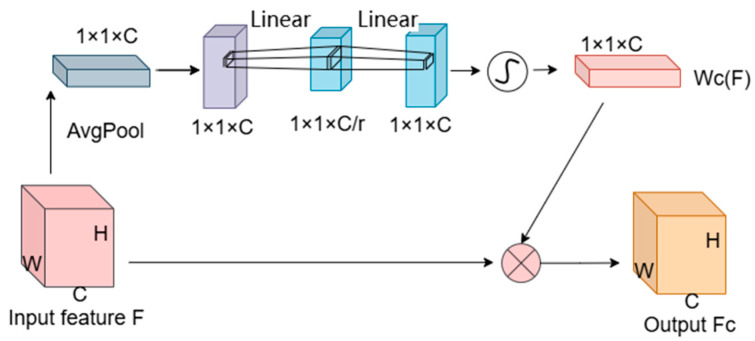
Structure of the Channel Attention Module.

The Hybrid Gated Attention (HGA) module initially employs global average and max pooling operations to extract global information from each channel, as depicted in Figure 4. This mechanism compresses the input feature map F with dimensions H × W × C to 1 × 1 × C via global average pooling, capturing global features for each channel. These features are then processed through a two-layer, fully connected network. The first layer reduces the dimensions to 1 × 1 × C/r, where ‘r’ is the reduction ratio, and applies the ReLU function for non-linear activation. Subsequently, the second fully connected layer restores the dimensions to 1 × 1 × C and employs a sigmoid function to generate the channel-specific adjustment weights $W_{C}(F)$. The final output, the channel-weighted feature map $F_{C}$, is produced by performing an element-wise multiplication of these weights with the original input feature map.

The process is described by the following equation:

(2) $$W_{C}(F)=\sigma (W_{2}\cdot \delta (W_{1}\cdot \mathrm{AvgPool}(F)))$$

(3) $$F_{C}=F\cdot W_{C}(F)$$

where F is the original input feature map, $W_{C}(F)$ denotes the channel attention map that reflects the outcomes of channel attention optimization, σ is the sigmoid function, δ is the ReLU activation function, $W_{1}$ and $W_{2}$ are the weights of the fully connected layers, AvgPool signifies global average pooling, and $F_{C}$ is the resulting channel-weighted feature map.

**Figure 5:**
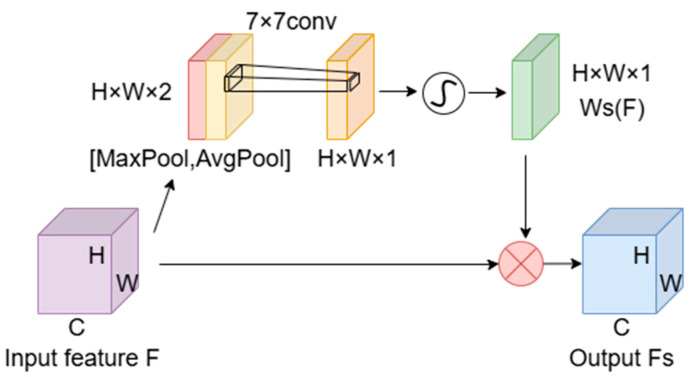
Structure of the spatial attention module.

As depicted in Figure 5, the spatial attention module initiates its process by separately applying max pooling and average pooling. Subsequently, it compresses the input feature map F along the channel dimension, yielding two distinct feature maps: an H × W × 1 max pooled feature map and an H × W × 1 average pooled feature map. These maps are then concatenated into a H × W × 2 feature map, which is processed through a 7 × 7 convolutional layer. The output from this layer, once passed through a sigmoid activation function, forms the spatial attention map $W_{s}(F)$. This map is scaled up to the dimensions of the original feature map F and is applied element-wise to F to produce the adjusted spatial attention feature map $F_{S}$.

The operation can be formulated as:

(4) $$W_{s}(F)=\sigma ({\mathrm{Conv}}_{7\times 7}(\mathrm{Concat}(\mathrm{AvgPool}(F),\mathrm{MaxPool}(F))))$$

(5) $$F_{S}=F\cdot W_{s}(F)$$

here, $W_{s}(F)$ denotes the spatial attention map that showcases the optimization results of spatial attention. MaxPool and AvgPool represent global max pooling and average pooling, respectively, facilitating the capture of comprehensive spatial features. Concat signifies the concatenation operation along the channel dimension, critical for integrating and processing spatial information. ${\mathrm{Conv}}_{7\times 7}$ specifies the convolutional operation using a 7 × 7 kernel size, while σ represents the sigmoid function that calculates the spatial attention weights, with $F_{S}$ being the resultant spatially weighted feature map.

To optimally integrate these attention modules, a gating mechanism has been incorporated. It dynamically adjusts their contributions to the final output by learning how to effectively merge these attention types. This dynamic adjustment allows the network to more effectively utilize various attentional cues, enhancing segmentation performance.

**Figure 6:**
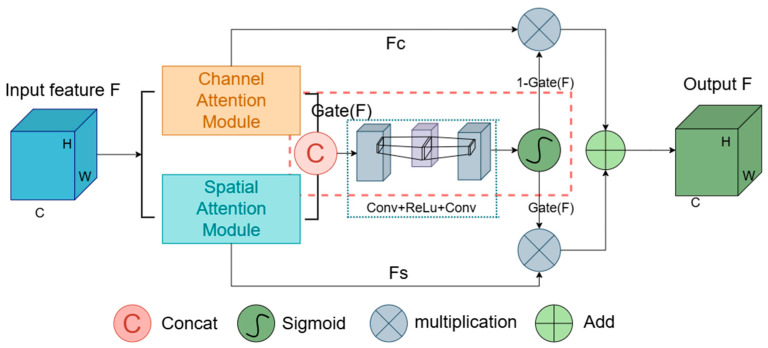
Structure of the Hybrid Gated Attention Module.

As illustrated in Figure 6, the gating mechanism initiates by applying attention weights to the input features, followed by concatenating the outputs of the channel and spatial attention modules along the channel dimension. This concatenated feature map undergoes processing through a 1 × 1 convolutional layer, reducing the number of channels by half or adjusting it to a predefined dimension. A ReLU activation function then adds non-linearity to the system. Another 1 × 1 convolutional layer further reduces the channel count, resulting in a single-channel output. This output is transformed by a Sigmoid activation function into a gating signal, dynamically adjusting the balance between channel and spatial attentions.

The operational formula is expressed as:

(6) $$\mathrm{Gate}(F)=\sigma ({\mathrm{Conv}}_{1\times 1}(\delta ({\mathrm{Conv}}_{1\times 1}(\mathrm{Concat}(F_{C},F_{S})))))$$

in this formula, Concat denotes the concatenation operation along the channel dimension, $F_{S}$ and $F_{C}$ are the spatially weighted and channel-weighted feature maps, respectively. ${\mathrm{Conv}}_{1\times 1}$ represent the spatially weighted and channel-weighted feature maps, respectively. δ and σ symbolize the ReLU and sigmoid functions, respectively, with the latter generating the final gating signal.

The final output is synthesized by blending the channel and spatial attentions through the calculated gating coefficient:

(7) $$F_{\mathrm{output}}=(1-\mathrm{Gate}(F))\cdot F_{C}+\mathrm{Gate}(F)\cdot F_{S}$$

the final output $F_{\mathrm{output}}$ is synthesized using the HGA gating mechanism, Gate(F) dynamically balances the contribution of spatial and channel attentions, where $F_{S}$ and $F_{C}$ represent the spatially and channel-weighted feature maps, respectively.

Within the RHEU-Net architecture, the Hybrid Gated Attention (HGA) module is integrated before the skip connections. This integration allows the model to capture both local details and global contextual information effectively before transmitting features to the decoder. This enhancement improves the precision of liver tumor segmentation. The HGA module optimizes the transmission of pivotal features and suppresses irrelevant feature interference. This method ensures that the features relayed to the decoder are highly informative, facilitating the model’s ability to intricately learn the multi-dimensional and irregular features of complex structures. Consequently, this enhances the model’s proficiency in accurately identifying the boundaries of the liver and its tumors.

### 3.3. Multi-Scale Feature Enhancement

In medical image segmentation tasks, multi-scale feature extraction and fusion techniques have always been key to enhancing model performance. GoogleNet utilizes its Inception module to capture information using convolutional kernels of various sizes simultaneously, thereby enhancing the network’s expressive power and improving the detail resolution of images [35]. SPPNet, through its Spatial Pyramid Pooling (SPP) technique, emphasizes efficient processing of inputs of different scales under a fixed output size, significantly reducing the computational burden of fully connected layers and preventing information loss [27]. However, using pooling to expand the receptive field can result in the loss of feature layer resolution. Inspired by SPP, DeepLabV3 employs Atrous Spatial Pyramid Pooling (ASPP) to further enlarge the receptive field, enabling more effective capture of extensive contextual information [28]. ASPP, as introduced in DeepLabV3 and inspired by SPP, utilizes multiple parallel atrous convolution layers with different sampling rates, processing features extracted at each rate in separate branches, and merging them to produce the final outcome. This module constructs convolutional kernels with various atrous rates to capture multi-scale object information, expanding the receptive field while also maintaining the resolution of feature layers [28].

**Figure 7:**
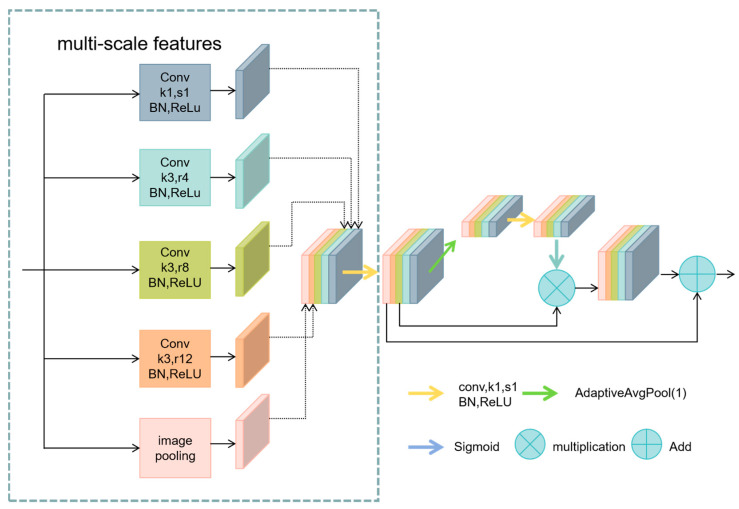
Structure of the Multi-Scale Feature Enhancement Module.

Inspired by these models and based on ASPP, the Multi-Scale Feature Enhancement Module (MSFE) module was designed, integrating ResNet architecture and feature enhancement strategies, and incorporated into the U-Net architecture. As depicted in Figure 7, the ASPP module comprises five parallel branches: a 1 × 1 conventional convolution layer, three 3 × 3 dilated convolution layers with dilation rates of 4, 8, and 12 to expand the receptive field, and a global average pooling layer to enhance global contextual information. The outputs of these five branches are concatenated along the channel direction and further integrated through a 1 × 1 conventional convolution layer.

In the MSFE module, dilated convolutions employ depthwise separable convolutions to extract spatial features independently from each input channel. These are then integrated, and inter-channel information is fused using pointwise (1 × 1) convolutions. This process reduces the number of parameters and computational complexity [36].

Following the extraction of multi-scale features, a global average pooling layer further extracts global information. The resulting pooled feature map is processed through a 1 × 1 convolution, batch normalization, and ReLU activation. This is followed by adaptive average pooling and Sigmoid activation to refine the features. This module computes an attention map, used to weight the original features, enhancing focus on significant features. It integrates attention-weighted features with the original concatenated features through skip connections, further enriching feature expression.

At the network’s bottleneck stage, where feature dimensions are significantly reduced, the network loses spatial information about the target of interest. In semantic segmentation, contextual information is crucial for accurately segmenting targets from input images [37]. Thus, the MSFE module replaces the original convolution blocks at the bottleneck layer, employing multi-scale feature extraction with multi-level sampling rates of dilated convolutions sampled in parallel to enrich semantic information and effectively capture global contextual information. By reweighting and optimizing the feature processing workflow, this approach mitigates potential local information loss due to dilated convolutions, suppresses irrelevant or distracting information, and provides rich semantic information for the decoding stage, thereby enhancing the accuracy and generalizability of segmentation tasks and compensating for the limitations of conventional convolutions when processing tumors of varying sizes.

### 3.4. Loss Function

The chosen loss function is BCEDiceLoss, which synergistically combines the benefits of Binary Cross-Entropy (BCE) and Dice losses. This approach integrates both, capitalizing on their strengths in pixel-level classification and overall segmentation quality.

Dice loss quantifies model performance by assessing the overlap between the predicted and actual segmentations. It is computed using the following formula:

(8) $$\mathrm{DiceLoss}=1-\frac{2\sum_{i=1}^{N}y_{i}\cdot x_{i}+\epsilon }{\sum_{i=1}^{N}y_{i}+\sum_{i=1}^{N}x_{i}+\epsilon }$$

where N denotes the total number of pixels in the sample, $y_{i}$ denotes the actual label value (0 or 1) of the ith pixel, $x_{i}$ is the predicted probability for that pixel, and ϵ is a small constant to prevent division by zero. However, reliance solely on Dice loss might not address some challenges encountered in liver tumor segmentation, such as when both the labels and the segmented regions are minimal, leading to large gradients that can destabilize the training process during backpropagation.

To compensate for these shortcomings, Binary Cross-Entropy (BCE) Loss is introduced:

(9) $$\mathrm{BCELoss}=-\frac{1}{N}\sum_{i=1}^{N}\left[y_{i}\cdot \log\left(x_{i}\right)+\left(1-y_{i}\right)\cdot \log(1-x_{i})\right]$$

where N denotes the total number of pixels in the sample, $y_{i}$ denotes the actual label value (0 or 1) of the ith pixel, $x_{i}$ is the predicted probability for that pixel. This component of the loss function imposes a penalty for misclassification at the pixel level, enhancing classification accuracy and providing stable gradients during training, thus facilitating model convergence.

The BCEDiceLoss is defined as the weighted sum of BCE and Dice losses:

(10) BCEDiceLoss=α⋅BCELoss+(1−α)⋅DiceLoss

where α is a weight parameter that balances the influence of the two losses. This balanced approach allows the function to capitalize on BCE loss for pixel-level accuracy and Dice loss for assessing the overlap between the predicted and true regions, thus ensuring an effective balance between precision and recall in the segmentation tasks. The loss is minimized, and optimal experimental results are obtained when α is set to 0.5.

## 4. Experiment Results

### 4.1. Experimental Configuration

The experimental environment was configured on an Ubuntu 20.04 system, utilizing a 14-core Intel Xeon Platinum 8362 processor, 45 GB of RAM, and an NVIDIA RTX 3090 GPU. Experiments were conducted using Python 3.8, PyTorch 1.10.0, and CUDA 11.3. We set the batch size to 4 and the initial learning rate to 1 × 10^−4^ for 120 epochs of training. The learning rate was adjusted using an exponential decay strategy with a decay rate of 0.95, and overfitting was countered with L2 regularization and Dropout.

### 4.2. Data

This research employs the publicly accessible LiTS2017 dataset, provided by the MICCAI 2017 Challenge [38], to validate the proposed segmentation algorithm. The dataset, sourced from six global medical institutions, consists of 201 abdominal enhanced CT scans using high-precision X-ray sensors for detailed imaging of liver structures and tumors. Of these scans, 131 are annotated for liver and liver tumors, and 70 are unlabeled. The dataset encompasses 58,638 CT slices, each at 512 × 512 pixels resolution [38]. Due to varied imaging equipment and protocols, the CT images exhibit notable variations in resolution and quality. The in-plane resolution varies from 0.55 to 1.00 mm, with the inter-slice resolution ranging from 0.45 to 6.00 mm. For this research, only the 131 annotated scans were used to build the dataset, with 30 randomly selected for the test set and the remaining 101 used for preprocessing.

### 4.3. Data Preprocessing

**Figure 8:**
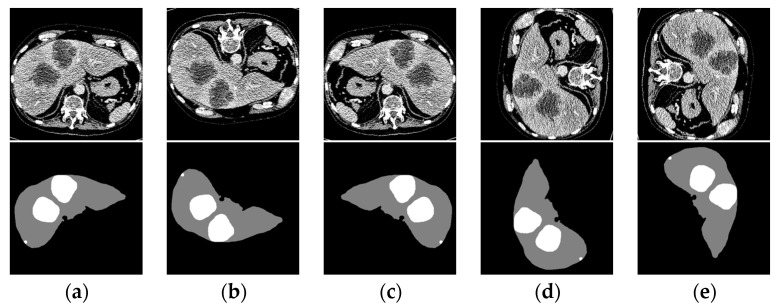
(**a**) Original image; (**b**) Flip horizontal; (**c**) Flip vertical; (**d**) Left rotation; (**e**) Right rotation.

The original CT images were initially randomly divided into training and validation sets in an 8:2 ratio. To improve image processing efficiency and accuracy, the sets were treated with windowing and Contrast Limited Adaptive Histogram Equalization (CLAHE) to adjust contrast, highlighting the liver region and minimizing noise [39]. The window settings were 200 HU for width and 40 HU for level. Additionally, the Min-Max normalization technique scaled the grayscale values to the [0, 1] range, aiding in further processing. Given the high number of slices in the original CT images, direct use could lead to excessive computational resource consumption. To address this, a 2.5D training strategy was implemented, using only the target slice and its two adjacent slices as inputs to reduce resource demand and improve prediction accuracy [40]. To combat overfitting, augmentation techniques such as random cropping and flipping were applied, with cropping adjusting the image size to (3 × 480 × 480). The processed image is shown in Figure 8.

### 4.4. Ablation Experiment

The RHEU-Net model, by integrating residual modules, hybrid gated attention modules, and MSFE, leverages multi-scale technologies and hybrid attention mechanisms to enhance its capability to extract contextual information. This integration improves the accuracy of segmenting liver edges and small tumors. To assess the specific contributions of these integrated modules to segmentation performance, this study employs the U-Net as a baseline model and conducted a series of ablation experiments focusing on liver and tumor regions. As per the segmentation data for the liver area presented in Table 1, the baseline U-Net model achieved a Dice coefficient of 94.27%. With the subsequent incorporation of residual modules, hybrid gated attention modules, and MSFE, the Dice coefficients increased by 0.58%, 0.91%, and 0.52%, respectively. Moreover, when hybrid gated attention modules and MSFE were sequentially introduced atop the residual modules, the Dice coefficients further rose by an additional 0.54% and 0.33%, respectively. The RHEU-Net model proposed in this research, which synergistically combines these three modules, realized a Dice coefficient enhancement of 1.45%, achieving the best performance across all evaluation metrics. This holistic integration not only highlights the effectiveness of each module but also demonstrates the model’s exceptional capability in segmenting liver regions.

According to the tumor segmentation data presented in Table 2, the baseline U-Net model achieved a Dice coefficient of 66.83%. With the integration of the residual module, hybrid gated attention module, and MSFE, the Dice coefficients improved by 1.48%, 1.83%, and 1.21%, respectively. Subsequent enhancements were noted when the hybrid gated attention module and MSFE were sequentially layered on top of the residual module, further raising the Dice coefficient by 1.16% and 0.72%. The modified RHEU-Net model, as proposed in this study, which incorporates all three aforementioned modules, demonstrated a significant increase in the Dice coefficient by 3.36%. It also performed optimally across other critical evaluation metrics, including the Jaccard index, precision, and recall. These findings not only confirm the efficacy of the individual modules but also underscore the RHEU-Net’s superior capability in segmenting tumor regions.

**Figure 9:**
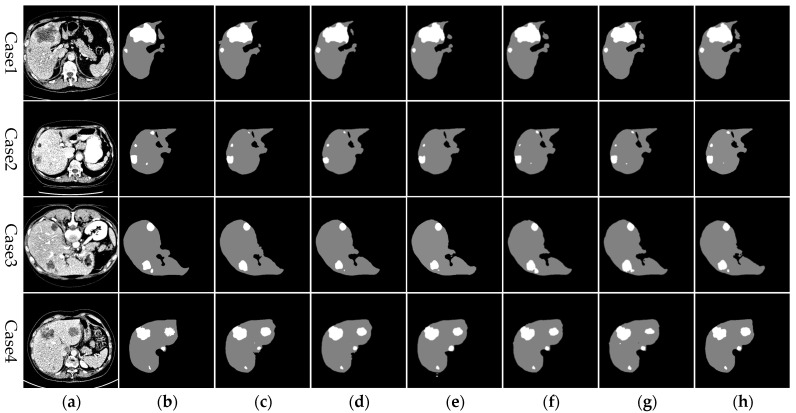
Segmentation results from various networks on selected test set images in the ablation experiment. From left to right: (**a**) original CT image, (**b**) gold standard, (**c**) U-Net, (**d**) Res+U-Net, (**e**) HGA+U-Net, (**f**) MSFE+U-Net, (**g**) Res+HGA+U-Net, and (**h**) RHEU-Net (method described in this study).

In the ablation studies, the test set was used to evaluate the segmentation performance of various network configurations, as shown in Figure 9. The results demonstrate that the addition of each module consistently enhances segmentation outcomes across both liver and tumor regions. With the progressive integration of modules, the delineation of liver and tumor boundaries becomes sharper, and the occurrence of over-segmentation and under-segmentation, especially in smaller tumors and liver sections, markedly diminishes, ultimately converging towards the quality of the gold standard. These findings affirm that RHEU-Net, with its integrated residual module, Hybrid Gated Attention module, and MSFE, effectively captures critical dimensional and spatial feature information, thus bolstering its capacity for contextual information extraction. Furthermore, the model refines the feature extraction process and reduces distractions from non-relevant areas, thereby enhancing the precision in identifying liver edges and small tumors, and consequently improving the overall segmentation accuracy for liver and liver tumors.

### 4.5. Analysis of Training Loss Rate

**Figure 10:**
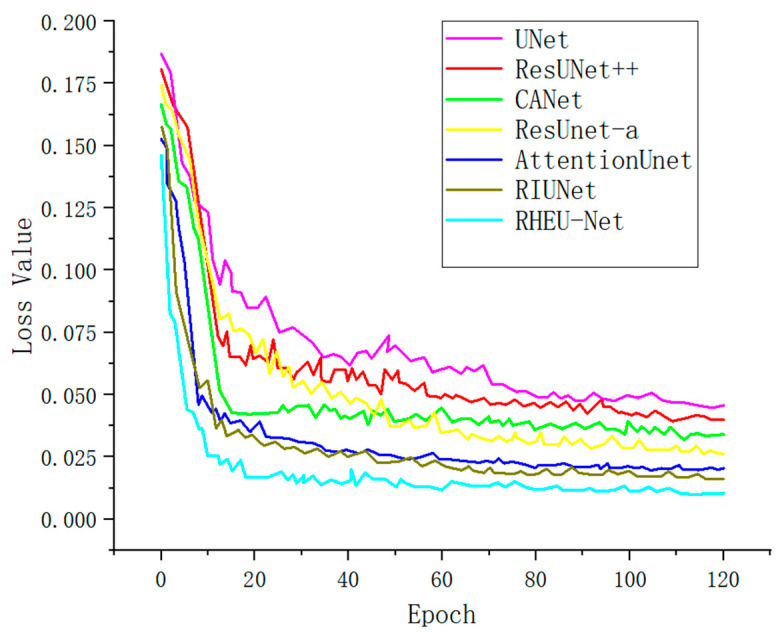
Training loss trends of different models.

Figure 10 vividly depicts the trends in loss values throughout the training process of various models. The x-axis represents the number of training epochs, while the y-axis denotes the models’ loss values. Initially, the loss values are high across all models as they have not yet fully adapted to the data patterns. With increasing iterations, each model undergoes optimization, resulting in a rapid decline and subsequent stabilization of loss values. It is evident that our model, RHEU-Net, exhibits substantial advantages in performance comparison. Its loss curve not only declines the fastest but also maintains the least fluctuation throughout the training process, demonstrating RHEU-Net’s excellent learning efficiency and high stability. These attributes underscore its superior network performance, making it outstanding in all evaluated performance metrics.

### 4.6. Liver Segmentation Results

To assess the liver segmentation efficacy of the proposed RHEU-Net approach, 30 CT images were randomly selected to form the test set. These images were used to benchmark the segmentation performance of RHEU-Net against four cutting-edge network models within this domain. The quantitative outcomes of these comparisons are presented in Table 3. The findings demonstrate that RHEU-Net outperforms six competing network models across several key metrics, including the Dice coefficient, Jaccard index, and precision. Although RHEU-Net slightly underperforms in recall compared to RIUNet and AttentionUnet, it nonetheless achieves the best composite performance on the other four critical metrics. In comparison to the conventional U-Net framework, RHEU-Net exhibits enhancements of 1.45% in the Dice score, 1.98% in the Jaccard index, 1.06% in precision, and 1.02% in recall. Relative to AttentionUnet, ResUnet-a, CANet, Res Unet++, and RIUNet, RHEU-Net’s Dice values increased by 0.49%, 0.59%, 0.86%, 1.23%, and 0.34%, respectively. These improvements underscore the network’s capability not only to elevate accuracy but also to effectively mitigate both false positives and false negatives, thereby enhancing the overall segmentation precision.

**Figure 11:**
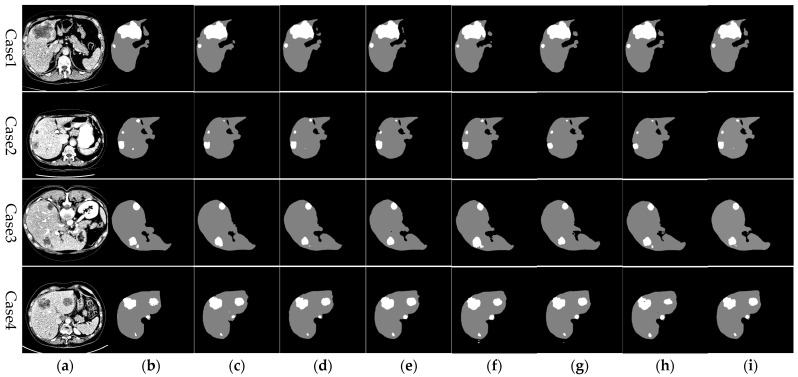
Comparison of liver segmentation results from different networks against the gold standard. From left to right, the images represent: (**a**) original CT image, (**b**) gold standard, (**c**) Unet, (**d**) AttentionUnet, (**e**) ResUnet-a, (**f**) CAUnet, (**g**) Res Unet++, (**h**) RIUNet, (**i**) RHEUnet (method described in this study).

Figure 11 illustrates the comparative analysis of liver segmentation outcomes from various network models against the gold standard. In these images, gray areas indicate the segmented liver regions, whereas white areas denote the tumor regions. Close examination of case 1 reveals that the RHEU-Net approach, as introduced in this paper, achieves more accurate segmentation of the smaller liver model in the upper right corner. Similarly, in cases 2 and 4, this approach more precisely captures the detailed liver contours, producing segmentation outcomes that more closely align with the gold standard, surpassing the performance of other models.

As depicted in Figure 11, other network models experienced issues of over-segmentation or under-segmentation during liver segmentation. This is primarily due to the weak and redundant shallow feature information extracted during the encoding phase, which adversely affects the segmentation outcomes. The method proposed in this paper introduces residual modules and hybrid gated attention modules (HGA) into the U-Net framework. These enhancements effectively capture crucial dimensional and spatial feature information during the segmentation process, addressing gaps in detailed information. Additionally, the use of MSFE in the bottleneck layer increases the receptive field of the convolutional kernels, thereby enhancing the network’s ability to extract contextual information. Moreover, by reweighting and optimizing the feature processing workflow, this approach alleviates potential local information loss caused by dilated convolutions and effectively suppresses irrelevant or distracting information, further improving the model’s ability to precisely predict liver edges.

### 4.7. Tumor Segmentation Results

Table 4 delineates the comparative segmentation results for liver tumor regions utilizing various network models. The innovative method introduced in this study surpasses six competing models on key metrics, including Dice coefficient, Recall, and Precision. Despite a marginally lower Jaccard index relative to AttentionUnet and RIUNet, this method showcases superior composite performance on the four other critical metrics. In comparison to the conventional U-Net network, significant gains are evident with a 3.36% increase in Dice score, a 4.44% enhancement in Jaccard index, a 3.04% rise in precision, and a 6.11% advancement in recall. Furthermore, against AttentionUnet, ResUnet-a, CANet, Res Unet++, and RIUNet, the Dice values improved by 0.54%, 3%, 2.36%, 2.74%, and 0.42%, respectively. These results underscore the method’s prowess not only in elevating segmentation precision but also in effectively capturing intricate details of liver tumors, thereby setting a new benchmark in medical image segmentation efficacy.

Figure 11 further displays the segmentation outcomes for liver tumors across various network models. The method introduced in this paper demonstrates superior tumor segmentation capabilities in cases 1, 2, 3, and 4, aligning more closely with the gold standard. In cases 2 and 3, U-Net, ResUnet-a, and Res Unet++ failed to detect small tumors, while Attention Unet managed to segment smaller tumors. In contrast, the proposed method accurately identified small tumors and achieved results more consistent with the gold standard. In case 4, U-Net and ResUnet++ incorrectly recognized parts of the liver and background regions as tumors. Meanwhile, the proposed method not only segmented larger tumor areas more completely but also effectively detected smaller tumor regions, mitigating issues of under-segmentation and over-segmentation to deliver results that more closely resemble the gold standard. These enhancements underscore the effectiveness of integrating residual modules, hybrid gated attention modules (HGA), and Multi-Scale Feature Enhancement (MSFE). This integration not only prevents gradient vanishing issues but also refines feature selection both channel-wise and spatially through the attention modules. Features are weighted according to their contribution to liver segmentation, enhancing feature expressiveness while expanding the receptive field. This optimized feature handling simultaneously suppresses interference from irrelevant areas, effectively capturing features of small tumors and enhancing tumor detection precision.

## 5. Discussion and Conclusions

This paper proposes RHEU-Net, a novel network designed for liver tumor segmentation. RHEU-Net incorporates multiple key design elements, enhancing the network’s feature extraction capabilities and improving gradient flow by integrating residual modules, which effectively mitigate the problem of gradient vanishing. By employing hybrid gated attention modules, the model achieves parallel processing of channel and spatial attentions, dynamically adjusts the feature fusion strategy, and precisely captures essential channel and spatial feature information, filling in missing details. This allows the model to effectively learn multi-dimensional and irregular features of complex structures. Additionally, Multi-Scale Feature Enhancement (MSFE) is used for multi-scale feature extraction, which not only expands the receptive field but also strengthens the extraction of contextual information, thereby enhancing feature representation.

To validate the effectiveness of RHEU-Net, we utilized high-resolution CT scan data as our training and testing sets. These data, sourced from CT scanners—advanced medical imaging sensors—provide images rich in depth and three-dimensional spatial information, which are crucial for accurately identifying liver edges and differentiating tumors from normal tissue. Ablation experiments conducted on the LiTS2017 dataset validated the effectiveness of the modules integrated into RHEU-Net. Comparative tests with other network models demonstrated that our proposed method outperforms the comparison models in liver segmentation with Dice coefficients, Jaccard, and Precision reaching 95.72%, 91.49%, and 96.23%, respectively. In tumor segmentation, the method also excelled in Dice coefficients, Precision, and Recall, achieving 70.19%, 85.45%, and 71.79%, respectively. Compared to the baseline U-Net, our method improved the Dice coefficients for liver and tumor segmentation by 1.45% and 3.36%, respectively. Although the network excels in segmenting the liver and its tumors, the difficulty of acquiring medical data remains a major challenge. In light of this, future research will focus on developing more efficient lightweight network architectures to better meet the rapidly evolving medical technology and demands [43,44]. In future research, we intend to explore the potential of RHEU-Net in expanding CT imaging workflows. We aim to extend its application beyond the current scope of liver segmentation. To achieve this, we plan to adjust RHEU-Net to optimize the processing of sensor-collected data. This will help enhance the model’s adaptability to various organs and different diagnostic requirements, thereby providing better support for physicians in their diagnostic practices [45].

## Figures and Tables

**Table 1:** Results of liver segmentation: ablation experiment.

Model	Dice	Jaccard	Precision	Recall
U-Net	94.27	89.51	95.17	94.96
Res+U-Net	94.85	90.49	95.74	95.65
HGA+U-Net	95.18	90.85	95.45	95.56
MSFE+U-Net	94.79	91.23	95.23	94.97
Res+HGA+U-Net	95.39	90.91	95.42	95.38
RHEU-Net(ours)	95.72	91.49	96.23	95.98

**Table 2:** Results of tumor segmentation: ablation experiment.

Model	Dice	Jaccard	Precision	Recall
U-Net	66.83	56.82	82.41	65.68
Res+U-Net	68.31	58.43	83.79	68.87
HGA+U-Net	68.66	58.25	84.83	68.95
MSFE+U-Net	68.04	58.17	83.06	68.89
Res+HGA+U-Net	69.47	60.12	85.24	70.62
RHEU-Net	70.19	61.26	85.45	71.79

**Table 3:** Results of liver segmentation experiment (bold is the best result of this indicator).

Model	Dice	Jaccard	Precision	Recall
U-Net [20] (2015)	94.27	89.51	95.17	94.96
AttentionUnet [22] (2018)	95.23	90.51	95.76	96.14
ResUnet-a [41] (2020)	95.13	90.24	95.65	95.94
CANet [42] (2020)	94.86	89.52	95.86	95.42
Res Unet++ [23] (2021)	94.49	89.64	95.84	95.29
RIUNet [40] (2022)	95.38	90.98	95.89	**96.23**
RHEU-Net(ours)	**95.** **72**	**91.** **49**	**96.** **23**	95.98

**Table 4:** Result of tumor segmentation experiment (bold is the best result of this indicator).

Model	Dice	Jaccard	Precision	Recall
U-Net [20] (2015)	66.83	56.82	82.41	65.68
AttentionUnet [22] (2018)	69.65	**61.43**	85.31	71.63
ResUnet-a [41] (2020)	67.19	57.92	85.03	66.58
CANet [42] (2020)	67.83	60.05	82.72	66.35
Res Unet++ [23] (2021)	67.45	59.49	82.28	65.82
RIUNet [40] (2022)	69.77	61.35	85.39	71.72
RHEU-Net(ours)	**7** **0.19**	61.26	**8** **5.45**	**7** **1.79**

## Data Availability

The public datasets analyzed during the current study are available in the LiTS repositories: https://competitions.codalab.org (accessed on 10 December 2023).
